# Spontaneous regression of hepatocellular carcinoma in a pure palliative care setting

**DOI:** 10.1002/jgf2.588

**Published:** 2022-11-09

**Authors:** Naoki Yamamoto, Chikara Yamamoto, Takeya Tajitsu

**Affiliations:** ^1^ Department of Home Care Shinsei Hospital Nagano Japan; ^2^ Department of Gastroenterology Hokushin General Hospital Nagano Japan

**Keywords:** hepatocellular carcinoma, palliative care, spontaneous regression

## Abstract

We report a case of spontaneous remission of hepatocellular carcinoma in an 84‐year‐old woman who was managed in our best supportive care clinic. The tumor, once relapsed regardless of the application of conventional transcatheter arterial chemoembolization and radiofrequency ablation, regressed spontaneously within 4 months. The presence of an occlusive thrombus in the portal vein feeding to the site of the tumor suggests that the reduced blood supply might have caused tumor necrosis. Furthermore, the successful eradication of hepatitis C virus maintained performance status, and good nutrition might play other roles on it.

## Graphical abstract

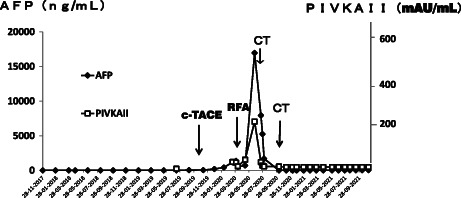

## 1 INTRODUCTION

Spontaneous regression of hepatocellular carcinoma (HCC) is very rare.^1^ It is estimated to occur in 1 in 140,000 cases worldwide.^1^ By 2019, only 106 cases were reported in the English written literature.^1^ We report a case of spontaneous regression of HCC in a woman with liver cirrhosis caused by hepatitis C virus (HCV) infection. The patient agreed to the publication of her case by providing informed consent. The project was approved by an institutional ethics committee. For human subjects, the investigation was conducted in accordance with the Declaration of Helsinki of 1975.

## 2 CASE PRESENTATION

**FIGURE 1:**
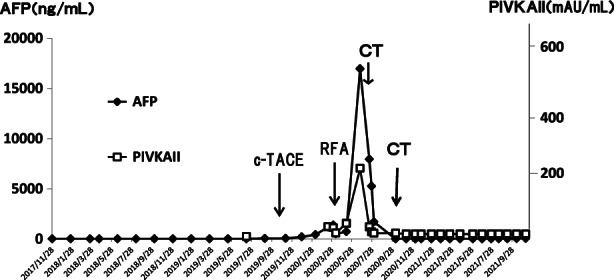
The patient's serum alpha‐fetoprotein and protein induced by vitamin K absence or antagonist‐II levels show a marked decline after discontinuing anti‐cancer therapy.

**FIGURE 2:**
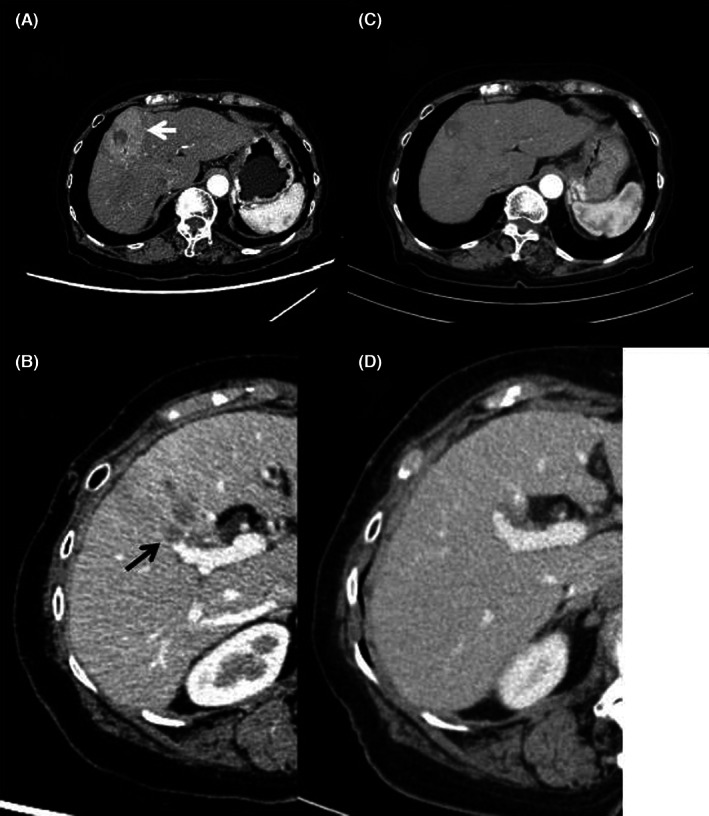
Axial image of contrast‐enhanced computed tomography shows a hypodense tumor with a diameter of about 3 cm in S8 (white arrow) (A) accompanied by portal vein thrombosis in P8 (black allow) (B) before regression in June 2020 and markedly decreased in size with the resolution of the portal vein thrombosis after regression in September 2020 (C, D).

An 84‐year‐old native Japanese woman presented to our clinic for the best supportive care (BSC) of HCC. She had been diagnosed with HCV‐related liver cirrhosis since 2008. A tumor 30 mm in diameter in segment 3 (S3) and a tumor in 10 mm in diameter in S8 developed as a complication of liver cirrhosis in 2017. She initially refused treatment, but she was treated with conventional transcatheter arterial chemoembolization (c‐TACE) of the tumor in S3 using a gelatin sponge and lipiodol combined with 20 mg of epirubicin on November 26, 2019. Subsequent c‐TACE of tumor in S8 was abandoned due to the patient's distress during the procedure. It was treated with radiofrequency ablation (RFA) on April 3, 2020. Ledipasvir 90 mg and sofosbuvir 400 mg were administered for 12 weeks in parallel with the tumor treatment resulting in the eradication of her HCV infection. Her serum alpha‐fetoprotein (AFP) and protein induced by vitamin K absence or antagonist‐II (PIVKA‐II) dropped once following the RFA, but they rose again persistently (AFP, 1377.7 ng/mL on April 1, 502.2 ng/mL on April 9, 731.3 ng/mL on May 11, 16962.3 ng/mL on June 22, PIVKA‐II, 45 mAU/mL on April 1, 24 mAU/mL on April 9, 62 mAU/mL on May 11, 282 mAU/mL on June 22 [Figure 1]), and on June 29, 2020, a recurrent tumor with a diameter of about 30 mm was found in S8, accompanied with an occlusive portal vein thrombus in P8 again (Figure 2). She was referred to our clinic on July 29, 2020, because she refused any more active anti‐tumor treatment. At the initial presentation, her Karnofsky Performance Status remained at 90. Laboratory examination showed serum albumin of 4.1 g/dL and total lymphocyte counts of 1615/mm^3^. On the contrary, serum AFP value had declined to 5261.8 ng/mL from 16962.3 ng/mL in 3 months. It continued to decline to 12 ng/mL 2 months later (Figure 1). When her PIVKA‐II was measured using the stored serum, it was found to have decreased simultaneously (Figure 1). Persistent eradication of HCV was confirmed using real‐time polymerase chain reaction. Contrast enhancement triple‐phase computed tomography was performed to confirm the spontaneous remission of her HCC. The previously affected area of S8 enhanced in the arterial phase with an early washout pattern in the portal phase was almost disappeared in the present computed tomography (CT). In addition, the portal occlusion on P8 had resolved (Figure 2). Giving the information about the spontaneous regression definitely pleased her and her caregivers. Her serum values of AFP and PIVKA‐II are gradually rising, but they remained still low over a further 1 year of follow‐up (AFP, 26.3 ng/mL; PIVKA‐II, 24 mAU/mL on December 13, 2021) (Figure 1). Throughout the entire time in the care of our BSC practice, she received neither curative treatment for cancer nor any specific treatment for symptoms due to the absence of discomfort.

## 3 DISCUSSION

The patient's clinical course is compatible with the definition of “spontaneous regression of cancer.”^2^

Tumor regression may be a delayed effect of RFA, but it is inconsistent with persistent elevation of tumor markers and CT findings for 2 months after RFA. Several hypotheses have been advocated to explain the mechanism of the spontaneous regression of HCC, but it has not been confirmed yet.^1^

Diminished blood supply by the thrombus in the portal vein should have caused our patient's tumor to become necrotic as previously reported.^1^ Six of the 24 spontaneous regression of HCC cases had impaired blood flow in the portal vein.^3^ Besides, one case of complete histopathological necrosis after preoperative portal vein embolization was reported.^4^ Recently, advances in molecular biology have led to the development of angiogenesis inhibitors that block the blood flow that cancer cells need to grow.^5^ Sorafenib, one of the angiogenesis inhibitors, has been used currently as a standard regimen for advanced HCC in Japan.^5^

Additionally, the successful eradication of persistent hepatitis viral infection which accelerates the carcinogenic process, maintenance of Karnofsky Performance Status, and good nutrition may have played other roles.^6^ With the accumulation of similar cases and integration of knowledge, we can expect to establish new treatment strategies for HCC.^1^, ^3^

A major limitation of this report is that we diagnosed the tumor as HCC based on laboratory data and imaging features^7^ without histopathological confirmation. However, it is well‐known that HCV infection is a major cause of cirrhosis, significantly increasing the risk of HCC.^8^ Given that similar cases have been reported previously,^1^, ^9^ we consider the clinical evidence to be sufficient.

In our case, further close follow‐up is required, as there have been some evidences that show once spontaneously regressed HCC cases recurred and resulted in death after few years.^1^, ^6^

We encountered spontaneous regression of HCC in the clinical setting of BSC. Spontaneous regression of cancer is a recognized verifiable phenomenon, even though it is quite rare.^2^ When caring for patients with advanced cancer, we must be more careful, not just obsessed with conventional concepts.

## AUTHOR CONTRIBUTIONS

All authors meet the ICMJE criteria for authorship.

## ETHICAL APPROVAL

None.

## PATIENT CONSENT

The patient has provided free written informed consent for the publication of this manuscript.

## CLINICAL TRIAL REGISTRATION

None.

## INFORMED CONSENT

The patient provided written informed consent.

## CONFLICT OF INTEREST

The authors declare no conflicts of interest for this article.

## PERMISSION TO PUBLISH

The patient has provided consent for the publication of this report.
